# Using surveillance data to monitor entry into care of newly diagnosed HIV-infected persons: San Francisco, 2006–2007

**DOI:** 10.1186/1471-2458-9-17

**Published:** 2009-01-14

**Authors:** Nicola M Zetola, Kyle Bernstein, Katherine Ahrens, Julia L Marcus, Susan Philip, Giuliano Nieri, Diane Jones, C Bradley Hare, Ling Hsu, Susan Scheer, Jeffrey D Klausner

**Affiliations:** 1 Division of Infectious Diseases, University of California, San Francisco, San Francisco, California, USA; 2 San Francisco Department of Public Health, San Francisco, California, USA; 3 School of Medicine, University of California, San Francisco, California, USA; 4 Positive Health Program, University of California, San Francisco, San Francisco, California, USA

## Abstract

**Background:**

Linkage to care after HIV diagnosis is associated with both clinical and public health benefits. However, ensuring and monitoring linkage to care by public health departments has proved to be a difficult task. Here, we report the usefulness of routine monitoring of CD4 T cell counts and plasma HIV viral load as measures of entry into care after HIV diagnosis.

**Methods:**

Since July 1, 2006, the San Francisco Department of Public Health (SFDPH) incorporated monitoring initial primary care visit into standard HIV public health investigation for newly diagnosed HIV-infected patients in select clinics. Entry into care was defined as having at least one visit to a primary HIV care provider after the initial diagnosis of HIV infection. Investigators collected reports from patients, medical providers, laboratories and reviewed medical records to determine the date of the initial health care visit after HIV diagnosis. We identified factors associated with increased likelihood of entering care after HIV diagnosis.

**Results:**

One -hundred and sixty new HIV-infected cases were diagnosed between July 1, 2006 and June 30, 2007. Routine surveillance methods found that 101 of those cases entered HIV medical care and monitoring of CD4 T cell counts and plasma HIV viral load confirmed entry to care of 25 more cases, representing a 25% increase over routine data collection methods. We found that being interviewed by a public health investigator was associated with higher odds of entry into care after HIV diagnosis (OR 18.86 [1.83–194.80], p = .001) compared to cases not interviewed. Also, HIV diagnosis at the San Francisco county hospital versus diagnosis at the county municipal STD clinic was associated with higher odds of entry into care (OR 101.71 [5.29–1952.05], p < .001).

**Conclusion:**

The time from HIV diagnosis to initial CD4 T cell count, CD4 T cell value and HIV viral load testing may be appropriate surveillance measures for evaluating entry into care, as well as performance outcomes for local public health departments' HIV testing programs. Case investigation performed by the public health department or case management by clinic staff was associated with increased and shorter time to entry into HIV medical care.

## Background

It is estimated that up to a third of HIV-infected patients aware of their infection are not receiving specialized HIV medical care in the U.S., and a similar proportion of those receiving care do not enter medical services until reaching a stage of advanced immunosuppression [1,2]. Clinically, early entry into care after diagnosis is associated with better health outcomes, as patients benefit maximally from timely initiation of highly active antiretroviral therapy (HAART), immunizations, and screening and prophylaxis for opportunistic infections and sexually transmitted diseases (STDs) [3]. Public health benefits of early linkage to care include access to risk-reduction interventions, possibly decreased HIV transmission due to reduced plasma and genital HIV viral load as a result of HAART, and possibly lower health care costs [3].

While case management and referral has been advocated as a method of decreasing unmet needs for supportive services and increasing utilization and adherence to HIV therapy [4,5], its role in linkage to care after HIV diagnosis remains poorly understood. At the public health level, collecting initial CD4 T cell counts and plasma HIV viral loads can determine the stage of HIV infection at diagnosis and serve as a measure of entry into medical care. Although several studies have used CD4 T cell count tests or plasma HIV viral load results as markers of care, those studies have not used provider or laboratory-reported test results as surveillance measures to assess entry to HIV medical care in the public health setting [2,5,6]. Surveillance of laboratory reports of CD4 T cell counts and plasma HIV viral loads could be used by public health departments as valid surrogates for entry into HIV medical care after diagnosis, allowing the design, evaluation, and improvement of HIV testing and linkage to care programs. Currently, few local public health departments are using CD4 T cell count data collected for these purposes and many states have not yet established mandatory laboratory reporting of CD4 T cell counts and plasma HIV viral loads.

Recently, the Centers for Disease Control and Prevention (CDC) prioritized linkage to care as a primary strategic prevention objective of the National Advancing HIV Prevention plan [3]. Current recommendations include the report of ongoing CD4 T cell count results to the local health jurisdiction, beginning at the time of HIV diagnosis [3]. CD4 T cell counts and plasma HIV viral load are used clinically to determine the stage of disease and indications for initiating antiretroviral therapy. Those tests are commonly obtained on the first visit for HIV medical care. Therefore, CD4 T cell counts and plasma HIV viral load could potentially serve as surrogate markers to evaluate entry into care, and determine unmet health needs in various communities.

In this report we describe the characteristics of patients having a newly diagnosed HIV infection between July 1, 2006 and June 30, 2007 who entered into HIV medical care. Secondly, we demonstrate the additional yield obtained by using laboratory surveillance of CD4 T cell tests and plasma HIV viral loads as markers for entry into HIV medical care after initial diagnosis. Lastly, we show the effect of having had a public health investigator interview after a first positive HIV test on the likelihood of entering medical care within 3 months of the initial HIV diagnosis.

## Methods

### Public health investigation and referral to HIV medical care

As part of its routine public health activities, the San Francisco Department of Public Health performed HIV public health investigation, including interview and referral to HIV medical care in all patients newly diagnosed with HIV infection at the San Francisco's municipal STD clinic, the San Francisco county hospital, and 13 community-based primary care clinics affiliated with the county public health care system. Activities and services included assuring test result disclosure; counseling, assessment and referrals for substance use, mental health, legal, disability, housing services; and voluntary third-party anonymous partner notification. Public health investigators made contact with patients through the telephone, letters, emergency contacts, and home or neighborhood/shelter visits. Patients were interviewed regarding demographics, prior and concurrent STDs, drug use, and sex partners and practices. Interviewed patients were referred to local HIV primary care providers, both within and outside of the San Francisco county public health care system. Patients were also assisted in making appointments and applying for medical insurance. All data were entered into electronic public health records.

### Documentation of entry into care

San Francisco has had name-based HIV reporting since April 2006. Laboratories are required by law to report all plasma HIV viral load test results to the health department. Additionally, CD4 T cell test results are collected by health department staff from major hospital laboratories in San Francisco or through medical records review. Since July 1, 2006, the SFDPH incorporated monitoring of the initial primary care visit into standard HIV public health investigation for newly diagnosed HIV-infected patients. We matched data on the public health investigation interview and referral to HIV medical care to the CD4 T cell counts and plasma HIV viral load reported to the county's HIV/AIDS Surveillance Registry using names and date of birth of patient. When discrepant laboratory results but same dates of specimen collection were found, we used the HIV/AIDS Surveillance Registry results. For this analysis we excluded persons with clinical HIV-related laboratory tests ordered on the same day of diagnosis (n = 26), assuming a high proportion of those cases may represent individuals with longstanding known infections who underwent HIV testing to document status for services. CD4 T cell counts or plasma HIV viral loads obtained at the emergency department or during acute hospitalization were not considered surrogates of entry to HIV primary care.

### Definition of outcomes

For the purpose of this study, entry into care was defined as having at least one visit to a primary HIV care provider after the initial diagnosis of HIV infection. Self-reports from patients and reports by medical providers were used to determine the date of the initial health care visit after HIV diagnosis. Date of first HIV diagnosis, date of first primary care visit, and date of first CD4 T cell and/or plasma HIV viral load test were used in our analysis to determine the time from diagnosis to entry into HIV care. We used first dates of HIV diagnosis and primary care visit.

### Statistical analysis

We used t-tests to compare days to entry into care between different subgroups and the Z-test to compare the proportions of cases who accessed care to cases who did not access care or for whom care status was unknown. We also identified factors associated with increased likelihood of accessing care after HIV diagnosis by using group-wise univariate odds ratios. To control for potential confounders, a logistic regression model was created and adjusted odds ratios (adjusted ORs) were determined. All variables included in the models were determined *a priori *based on epidemiological importance and biological plausibility. The initial model included the following variables: sex, sexual orientation, age category, site of testing, interviewed by the SFDPH, race/ethnicity, and CD4 T cell category. However, age, race/ethnicity, sex and sexual orientation were not found to contribute significantly to the model (p > .05) and were excluded from the final model. We used STATA/IC version 10.0 (StataCorp Inc, College Station, Texas) and SAS version 9.1 (SAS Institute, Cary, North Carolina) for the data analyses. The University of California San Francisco Committee on Human Research approved this study and waived patient consent requirements.

## Results

Between July 1, 2006 and June 30, 2007, 186 newly HIV-infected patients were diagnosed at San Francisco's municipal STD clinic, the San Francisco county hospital, and affiliated community-based primary care clinics. Of these, 26 had CD4 T cell counts and plasma HIV viral loads ordered on the day of diagnosis and were excluded from further analysis. Of the remaining 160 HIV-infected patients, 150 (95%) were men or male-to-female transgender; 124 (78%) reported having sex with men; 74 (48%) were white, 38 (25%) were Hispanic, and 29 (19%) were African-American. The median age was 35 years (range 18 to 81 years). Fifty-seven (36%) cases were diagnosed by rapid HIV test and 103 (64%) by standard HIV test; 79 (49%) were diagnosed in 2006 and 81 (51%) in 2007; 106 (66%) were diagnosed at San Francisco's municipal STD clinic and 54 (34%) at the San Francisco county hospital and affiliated clinics (Table 1).

**Table 1:** Characteristics of newly diagnosed HIV-infected cases with confirmed entry into care, San Francisco Department of Public Health, July 2006 to June 2007 (N = 160)

	**Confirmed** **(n = 126)**	**Not in care ** **or unknown ** **care status** **(n = 34)**	**P value**	**Univariate ** OR	**P value**	**Multivariate ** OR	**P value**
**Sex**							
**Male**	117 (93%)	34 (100%)	NS	0.30 (0.04 – 2.56)	0.28		
**Women**	9 (7%)	0	NS				

**Self-reported sexual orientation**							
**Men who have sex with men**	93 (74%)	31 (91%)	0.05	0.22 (0.07 – 0.66)	<0.01		

**Race/ethnicity**							
**White**	61 (48%)	13 (38%)	NS				
**Black**	22 (17%)	7 (21%)	NS	1.22 (0.48 – 3.11)	0.68		
**Hispanic**	30 (23%)	8 (24%)	NS	1.14 (0.49 – 2.64)	0.76		
**Asian**	7 (6%)	4 (12%)	NS	0.81 (0.22 – 3.02)	0.76		

**Age categories**							
**Less or equal to 25 years**	22 (17%)	12 (35%)	NS				
**26 to 35 years**	39 (31%)	11 (32%)	NS	1.32 (0.53 – 3.27)	0.56		
**36 to 45 years**	45 (36%)	7 (21%)	NS	1.68 (0.67 – 4.23)	0.27		
**Older than 45 years**	20 (16%)	4 (12%)	NS	1.86 (0.59 – 5.89)	0.29		

**Site of diagnosis**							
**Municipal STD clinic**	74 (59%)	32 (94%)	<0.01				
**County hospital**	37 (29%)	0	<0.001	27.6 (3.64 – 208.84)	0.001	101.71 (5.29 – 1952.05)	<0.001
**Community clinics**	12 (10%)	1 (3%)	NS	9.2 (1.15 – 73.34)	0.04	18.53 (0.75 – 457.98)	0.07
**Other**	3 (2%)	1 (3%)	NS	2.3 (0.23 – 22.84)	0.48		

**Initial CD4 T cell count**							
**Less or equal to 200**	30 (19%)	NA	NA				
**Between 200 and 500**	21 (13%)	NA	NA	7.05 (2.80 – 17.71)	<0.001	5.99 (1.75 – 20.44)	<0.01
**Higher than 500**	66 (48%)	NA	NA	13 (3.66 – 46.15)	<0.001	7.61 (1.71 – 33.75)	

**Interviewed by the SFDPH**	88 (70%)	33 (97%)	NS	1.86 (0.87 – 3.94)	0.11	18.86 (1.83 – 194.80)	0.001

**First primary care visit within 3 months of diagnosis**	111 (88%)	NA	NA	20.88 (8.85 – 49.25)	<0.001	7.44 (2.45 – 22.57)	<0.001

NS = Not significant, NA = Not applicable

Of the 160 newly HIV-infected cases included in the analysis, 121 (76%) were interviewed by a public health investigator. One hundred and one (101 (63%) out of 160 newly HIV-infected cases) patients were initially reported to enter HIV medical care after diagnosis by self-report or by a health provider. Laboratory records of CD4 T cell counts or plasma HIV viral loads corroborated the entry into HIV care of all (100%) of those 101 patients. In addition, 25 cases were confirmed to have entered HIV medical care by having records of CD4 T cell counts or plasma HIV viral loads, representing a 25% increase from self-report or report by a health provider. Out of those 126 cases with confirmed entry into care, 111 (88%) accessed care within the first 3 months after diagnosis (Figure).

The average value of the first CD4 T cell count was significantly different between cases diagnosed at San Francisco's municipal STD clinic and the San Francisco county hospital (487.9 cells/mm^3 ^[CI, 412.1–563.8] vs. 288.4 cells/mm^3 ^[CI, 202.8–373.9]). After adjusting for confounders we found that being interviewed by a public health investigator and being diagnosed at the San Francisco County hospital or community-based primary care clinic were strongly associated with entry into care after diagnosis (Table 1). Age older than 30 years was also associated with accessing HIV care within the first 3 months after diagnosis; however, it did not reach statistical significance (Table 1).

## Discussion

California requires laboratory reporting of plasma HIV viral load test results. The San Francisco Department of Public Health acquires CD4 T cell count information through medical record review and voluntary laboratory reporting. By routine surveillance of CD4 T cell counts and plasma HIV viral loads on patients newly diagnosed with HIV infection, we were able to document linkage to care in 25% more patients than by patient or provider report. Similarly, time to accessing care after HIV diagnosis was documented in those patients. Consistent with prior studies suggesting that a significant proportion of newly diagnosed HIV-infected patients are diagnosed late in the course of their disease, 19% of the HIV-infected cases who had CD4 T cell counts and plasma HIV viral load available in our sample had a first CD4 T cell count consistent with an AIDS diagnosis [1].

About 20–30% of HIV-infected patients who eventually enter care have a delay in entering care [2]. However, outreach and public health investigation has been found to increase the likelihood of entering and maintaining primary care [4,6,7]. In our sample, 79% (126 out of 160 newly diagnosed HIV-infected cases) of cases had confirmed entry into HIV care after diagnosis and 88% (111 out of 126 cases with confirmed entry into care) entered care within the first 3 months after diagnosis. We found that newly HIV-infected patients interviewed by public health investigators were more likely to access HIV medical care after diagnosis compared to non-interviewed cases. Interviews and investigations performed by public health staff led to an increased yield in the report of CD4 T cell counts and plasma HIV viral loads when compared to HIV/AIDS Surveillance Registry laboratory reports alone.

Importantly, and contrary to prior studies, we found that being diagnosed at the San Francisco county hospital and other community-based primary care clinics was strongly associated with entering care after HIV diagnosis when compared to patients diagnosed at the San Francisco municipal STD clinic [5,8]. Although the reasons for this finding remain unclear, it may reflect various factors including patient characteristics, the medical setting, and factors related to reasons for HIV testing. In addition, the San Francisco county hospital has initiated a multidisciplinary program to assure that all HIV-infected patients diagnosed at the hospital receive intensive care and public health investigation. Although the design of this study does not allow exploration of the effect of that hospital-based program on linkage to care, it seems likely that it played a major role in the success achieved by that site.

Several limitations of our findings should be acknowledged. The purpose of our study was to describe the characteristics of newly diagnosed cases of HIV infection and the usefulness of CD4 T cell counts and plasma HIV viral loads as surrogate measures for entry into care after the initial HIV diagnosis. Therefore, we excluded patients who had CD4 T cell counts and plasma HIV viral loads on the same day of HIV diagnosis, assuming that these may represent individuals with longstanding infections who underwent HIV testing to document status for services. Although it seems unlikely, by excluding these patients we might have excluded a group of patients who were linked to care on the same day of HIV diagnosis. Given that we defined entry into care as having had at least one visit to an HIV-care provider, this study does not allow any conclusions regarding maintenance of care after the initial visit. The use of multiple approaches to determine our primary outcome (entry into care) might have introduced biases to the analysis. Information provided by patients and health care providers interviewed by public health investigators are subject to social desirability and recall bias. Due to the observational nature of this study, our conclusions are subject to the limitations of observational studies and we cannot infer causality. Inaccuracy and incompleteness of documentation in the patients' charts and laboratory records, delayed reporting in surveillance data, as well as variation in the efforts invested by different public health investigators to obtain those data might have decreased the accuracy of our data or introduced biases.

## Conclusion

With the advent of expanded HIV testing, initial CD4 T cell count and plasma HIV viral load surveillance data can help local health departments determine when HIV-infected persons are being linked to care and the stage of HIV infection at the time of diagnosis. The time from HIV diagnosis to initial CD4 T cell count and plasma HIV viral load may be an appropriate surveillance measure for marking entry into care, as well as a performance measure for public health departments' HIV prevention and testing programs.

## Competing interests

In the past 12 months Dr. Klausner has received funding for research or educational purposes from Gen-Probe, Inc., and Gilead Sciences, Inc. Other authors report no potential conflict of interests.

## Authors' contributions

NMZ, KB, SP, LH, SS and JDK conceived of the study, participated in its design and coordination, and helped to draft the manuscript. NMZ, KB, KA and JM performed the statistical analysis, interpreted the results, and helped draft the manuscript. SP, GN, DJ, CBH and LH coordinated the data collection and made substantial contributions to the interpretation of data. All authors read and approved the final manuscript.

## Pre-publication history

The pre-publication history for this paper can be accessed here:


